# A Bowling Exergame to Improve Functional Capacity in Older Adults: Co-Design, Development, and Testing to Compare the Progress of Playing Alone Versus Playing With Peers

**DOI:** 10.2196/23423

**Published:** 2021-01-29

**Authors:** Jorge Luiz Andrade Da Silva Júnior, Daiana Biduski, Ericles Andrei Bellei, Osvaldo Henrique Cemin Becker, Luciane Daroit, Adriano Pasqualotti, Hugo Tourinho Filho, Ana Carolina Bertoletti De Marchi

**Affiliations:** 1 School of Physical Education and Physiotherapy University of Passo Fundo Passo Fundo Brazil; 2 Institute of Exact Sciences and Geosciences University of Passo Fundo Passo Fundo Brazil; 3 School of Physical Education and Sport of Ribeirão Preto University of São Paulo Ribeirão Preto Brazil

**Keywords:** functional status, elderly, virtual reality therapy, user-centered design, software design, video games

## Abstract

**Background:**

Older people often do not meet the recommended levels of exercise required to reduce functional decline. Social interaction is mentioned by this cohort as a reason for joining group-based exercises, which does not occur when exercising alone. This perspective shows that exergames can be used as motivational resources. However, most available exergames are generic, obtained from commercial sources, and usually not specifically designed or adapted for older people.

**Objective:**

In this study, we aim to co-design and develop a new exergame alongside older participants to (1) tailor the game mechanics and optimize participants’ adherence to and enjoyment of exercise; (2) test the participants’ functional capacity, motivation, and adherence to the exergaming program; and (3) compare these scores between those who played alone and those who played with peers.

**Methods:**

We conducted a co-design process to develop a new exergame adapted to older people. For user testing, 23 participants were divided into 2 groups to play individually (alone group) or to compete in pairs (with peers group). They played the game twice a week, resulting in 21 exergaming sessions. We assessed the participants’ General Physical Fitness Index (GPFI) before and after the user testing. We also administered questionnaires about the gaming experience and exercise adherence with its motivators and barriers.

**Results:**

We introduced a new bowling exergame for Xbox with a Kinect motion sensor that can be played in single or multiplayer mode. For the GPFI measurements, the sample was homogeneous in the pretest (with peers group: mean 40.5 [SD 9.6], alone group: mean 33.9 [SD 7.8]; *P*=.11). After the exergame testing sessions, both groups had significant gains (with peers group: mean 57.5 [SD 8.7], *P*=.005; alone group: mean 44.7 [SD 10.6]; *P*=.02). Comparing the posttest between groups, it was found that the group in which participants played with peers had better outcomes than the group in which participants played alone (*P*=.02). Regarding the gaming experience and exercise adherence, both groups recognized the benefits and expressed enthusiasm toward the exergame.

**Conclusions:**

The findings suggest that the developed exergame helps in improving the functional capacity and adherence to physical exercise among older people, with even better results for those who played with peers. In addition to leading to more appropriate products, a co-design approach may positively influence the motivation and adherence of participants.

## Introduction

Functional capacity expresses an individual’s capacity to perform submaximal activities [1]. For older people, decreased functional capacity may negatively influence the performance of daily activities and everyday life, interfering with the person’s independence, which is directly associated with impairments in functional performance [2,3]. To reduce functional decline, health professionals recommend physical activity, as it positively influences physical aptitude, cognition, and overall health conditions [4]. Frequent and regular physical exercise is an appropriate recommendation, as it can prevent numerous age-related declines, improve functional capacity, and improve quality of life [5,6]. However, despite the advantages of exercise routines, physical inactivity is highly prevalent among older people, and they might find it difficult to meet the minimum recommended activity levels needed to maintain health benefits [7-9].

The search for appropriate and attractive ways to engage older people in physical exercise is a challenging process [10]. In this perspective, exergames are presented as safe and beneficial resources for the practice of physical exercises by older people [11,12]. This type of game uses motion sensors to capture the player’s actual movements, stimulating physical interaction [13]. Exercise routines based on digital games are promising to keep players physically and socially motivated [14-16]. In addition, they present motivational factors such as performance evaluation and feedback, which may reduce dropout rates [17,18]. However, a considerable part of the available exergames is from commercial sources, generally not designed exclusively for older people or based on specific protocols [19]. Furthermore, a generic approach is not the best solution to engage older people; thus, exergames need to be personalized to match their goals and performance levels [11].

Studies indicate that the practice of physical activity when performed alone does not show good adherence by older people because of the lack of motivation [20,21]. In contrast, social interaction is the central aspect mentioned by older people as a reason for joining group exercises [22-24]. Practices that combine the advantages of group-based exercise instructed by a professional can increase effectiveness through a personalized program with specific exercise routines, keeping older people motivated to exercise [10,25]. Socializing, either through cooperation or competition, has been investigated as a motivational factor mentioned by players who prefer exergaming with peers [26,27].

Although several studies report that exercising with other people influences the motivation of older people, further research is needed to verify whether the practice of exergaming in the same conditions as the conventional exercise may increase adherence. In addition, studies should verify whether exergames can positively affect the health outcomes of participants. We assumed that an exergame designed specifically for older people could produce better outcomes and engagement. Before implementing an exergame, the needs, wishes, and motivation of the intended players should be assessed, for example, by applying user-centered design methods [11]. Moreover, different processes and activities should be considered to increase engagement in the exergame [11]. A co-design process can significantly contribute to product development, reducing social stigma, empowering users [28], and giving voice to patient groups in the creation process [29]. This study has 3 objectives. First, we intended to co-design and develop a new exergame alongside older participants to tailor the game mechanics and optimize their adherence and enjoyment for exercise. Thereafter, we tested the participants’ functional capacity, motivation, and adherence to the exergaming program and compared these scores between those who played alone and those who played with peers.

## Methods

### Recruitment and Participants

We conducted a co-design process and tests with 23 older participants who attended Caixeiral Campestre Club, based in the city of Passo Fundo, Rio Grande do Sul, Brazil. The inclusion criteria were as follows: participants who reported practicing some type of physical activity at least once a week before this study and who agreed to pause it during the testing period. All participants also had to have prior knowledge about the use of any type of technology, such as smartphones, computers, or video games. Men and women older than 60 years were accepted if they had cognitive aptitude attested by the Mini-Mental State Exam [30], which we administered before the intervention. As exclusion criteria, we excluded participants who reported having been discouraged by a physician to practice physical activity, had been hospitalized in the past 20 days, or would not have been able to attend all gaming sessions. Written informed consent was obtained from all participants. The local ethics committee of the University of Passo Fundo approved all procedures involving human participants under the opinion number 2.784.343.

For the test procedure, participants were separated into 2 groups: the *alone group*, initially with 11 participants who played the exergame individually, and the *with peers group*, initially with 12 participants who played the exergame, competing in pairs. Participants were allocated to the groups on an alternate basis by the order of agreement to participate. Throughout the tests, the pairs could watch each other play. As they were colleagues at the club, they also frequently talked about the game even outside the test sessions.

### Game Co-Design and Development

The development team consisted of 3 fitness trainers, 2 software developers, and 3 human-computer interaction specialists with prior experience in game development for older people. A multidisciplinary team was fundamental for project development, encouraging communication between professionals and participants. The human-computer interaction specialists assisted in enrolling participants for the co-design practice and specifying aspects of game interaction such as esthetics, gameplay, levels, scoring, competition, and other features. Fitness trainers explored the possibilities of training exercises, assisted with gameplay orientations, and assisted with the levels of difficulty, according to the body movements required to complete the actions in the game.

Our study was guided by 3 main aspects, which examined the interface design, game design, and player experience [31]. For game design and development, we applied a co-design process using experience-based design theory, based on the method described by Askenäs et al [32]. We also followed the guidelines formulated by Planinc et al [33], which consider the development of exergames controlled by body movements designed especially for older players. The guidelines include the use of appropriate gestures, minding the physical condition of the participants and avoiding small objects. Planinc et al [33] also mentioned the encouragement of social interaction and that the exergame should be adjusted to the interests of older people, based on activities that are familiar to them.

Following the co-design actions, in the first stage of the process, the development team had 6 meetings to discuss aspects related to technology, game design, testing, purpose of the study, and expected results. The meetings were held once a week for approximately 50 min at the club where the tests would be performed. In the meetings, other exergames developed and practiced specifically by older people were investigated to ascertain what aspects and characteristics should be incorporated in the development of a game for this target audience.

In the second stage, we set up the co-design group, which included the development team and members of the club who were willing to participate in the design process. The contributions of the other team members helped encourage the user participants to join the group. All participants met at the same location and were introduced to each other and the objectives of the study. The pace of the meetings was controlled by the researchers to ensure that the process remained on track and that the procedures were fully understood by all participants. During 2 meetings, the group brainstormed ideas to discuss the types of games that older people would like to play. The team of professionals provided insights on the choice of gaming, each according to their expertise. The user participants suggested that they would like to play bowling, because of its similarity to the real-world game, that is, the way of playing was already familiar to them. They also stated that they would enjoy playing virtual bowling because it reminded them of the activity they practiced with family and friends.

The software developers believed that they could implement socializing and competition features in the bowling game. The fitness trainers affirmed that bowling was a suitable choice as the movements performed by the player are the same in the real and virtual world. Therefore, all members of the group agreed that bowling was the most appropriate choice of game. Bowling is a closed skill sport that requires fine-tuning and training of self-paced predefined movements as well as a high level of concentration and balance to completely knock down the pins [34-36]. This kind of game may also help prevent an inactive life, promote physical and cognitive functions, and result in entertainment and motivation with social presence [6]. Furthermore, bowling allows slow movements that correspond to typical physical activities that are recommended for older people [37]. In this sport, they can practice by performing body movements such as real sports where flexibility, abductions, and extensions of the lower and upper limbs are required.

In the third stage, the group described the main tasks, activities, requirements, and objectives that they believed should be a part of the game. The tasks were designed to be simple in structure to facilitate user understanding and attention span. Some exergaming concepts were analyzed, and a few design ideas were tested. After this stage, possible design solutions were elaborated, building ideas on how the game could be presented to users, from sketches following the overall concept and the information collected in the previous stages. Participants could freely explore and express their thoughts. No detailed models were expected as the data collection method was new to the user participants.

The game was implemented using a Kinect v2 motion sensor (Microsoft Corporation) and motion capture technology to guide and immerse the avatar in a 3-dimensional (3D) environment. The game is compatible with devices running the Windows operating system (Microsoft Corporation). We used Unity 3D (Unity Technologies) as the game engine, Blender (Blender Foundation) for modeling and texturing the game objects, and Adobe Fuse (Adobe Inc) for the creation and modeling of the 3D characters. When the first stable version of the game was concluded, we began the tests. Throughout the testing period, the group reported feelings and impressions about the game, including difficulties, problems, and improvements that could be implemented. The technique used to gather information at this stage was observation and the think-aloud method to collect the participants’ immediate feedback [38]. All collected information was implemented during the testing period. As new characteristics were implemented, the game was updated for the sessions with the user participants. This process is characterized as iterative, in which activities and assessments must be repeated until a satisfactory solution is found [39]. The iterative development process helped researchers reliably collect tangible data. At the end of this study, everyone who was a part of the research met in a focus group to ensure that the results met the genuine needs of older people and to clarify previous experiences and impressions, considering each member’s vision and level of satisfaction with the game developed.

### Outcome Measures

We used the following measurements to assess participants and their interaction with the game:

- The Senior Fitness Test [40] was used to assess functional capacity and obtain the General Physical Fitness Index (GPFI). It consists of 6 tests designed to assess physiological parameters associated with independent functioning: lower and upper body strength, aerobic endurance, lower and upper body flexibility, and agility and dynamic balance. Numerous studies have shown that functional capacity can be assessed with several instruments to provide important diagnostic and prognostic information in a wide variety of clinical and research settings [1].
- With the Physical Exercise Adherence Questionnaire [41], we used the 13 yes or no statements of motivators and 12 yes or no statements of barriers for exercising, in this case, related to the exergame. Picorelli et al [41] developed and validated this questionnaire in Portuguese, which was adapted for the Brazilian older adult population, considering Brazil’s cultural, economic, and social contexts.
- Game Experience Questionnaire (GEQ) [42] was used to characterize players’ gaming experience during and after the gaming session. We used its in-game and postgame modules, which contain 14 statements and 17 statements, respectively, with a semantic differential scale response ranging from 0 to 4. Each component score is computed as the average value of its specific items. GEQ has been widely used in traditional game studies [43] and studies dealing with exergames including older participants [44].

### Testing Procedure

In the pretest, participants were interviewed in a predetermined setting on the club premises. On that occasion, a fitness trainer administered the Senior Fitness Test to all participants. In the test period, participants from both groups played the exergame in sessions twice a week, resulting in 21 exergaming sessions. This twice-weekly design and the 10-week duration were defined based on an average of similar studies [3,10]. Each participant had 10 rounds per session, with 2 throws each, regardless of whether the player scored points. There were 10 throws with the right arm and 10 throws with the left arm. Therefore, each participant had 20 throws, resulting in approximately 10 min of gameplay per session. A fitness trainer followed all sessions and instructed participants on the gameplay rules. For the with peers group, the throws were interspersed between the 2 participants of the pairs, who played competing and seeing the opponent’s score on the game screen. During the testing period, participants were not allowed to perform any other type of physical exercise. After the gaming test period, all participants had their functional capacity reevaluated using the Senior Fitness Test. The participants also answered the GEQ and Exercise Adherence Questionnaire.

### Statistical Analysis

Quantitative data were analyzed using the SPSS 22.3 statistical package (IBM Corp). We performed Wilcoxon signed intergroup comparisons based on negative posts to obtain the GPFI through the Senior Fitness Test for pre and posttest in both groups. We also performed Mann-Whitney tests with signed intragroup comparisons. We performed Mann-Whitney *U* test with signed intragroup comparisons to analyze the GEQ scores. All analyses considered a 95% CI (significance for *P*≤*.*05).

## Results

### Game Evolution

We intended to design a playful experience in which the physical movements themselves would contribute to the meaning of the game. The first contact participants had with the game was with a simpler first version, which had only one character and no sound effects. In this version, the game consisted of 2 distinct scenarios, one with the main menu and one with the bowling alley and surrounding bleachers, both with simple and homogeneous textures. The menus and the scoreboard of the rounds had only basic features.

During the testing period, configurations and improvements were implemented gradually, according to the need observed in the interactions through the participants’ reports. The game scenario was set with realistic texturing. Background songs were added to the home menu and for the game rounds. Rolling ball and falling pins sound effects were also added. Later, we included the option to return to the menu during the game without closing it. In that version, a female avatar was included, as the participants questioned the absence of such a character. In the menu implementation, we added the input of the participant’s name, gender, and age as well as a ranking with all registered players’ scores. Various colors and textures of bowling balls were also added and randomly chosen when the participant had to catch the ball to throw. In that version, the ball was set at a variable speed, according to the player’s throwing strength.

For the intermediate version, the characters were replaced by 6 new ones, 3 from each gender. The characters resembled older people. The bleachers were composed of a cheering audience to motivate the participants. We updated the instance system for balls, pins, scores, and match history. The pin counting and replacement system were simplified, and the balls on the rack were no longer static as before, as they had to be instantiated and rolled through the rack to be released. The participant could choose the character and start the round using an arm gesture.

**Figure 1:**
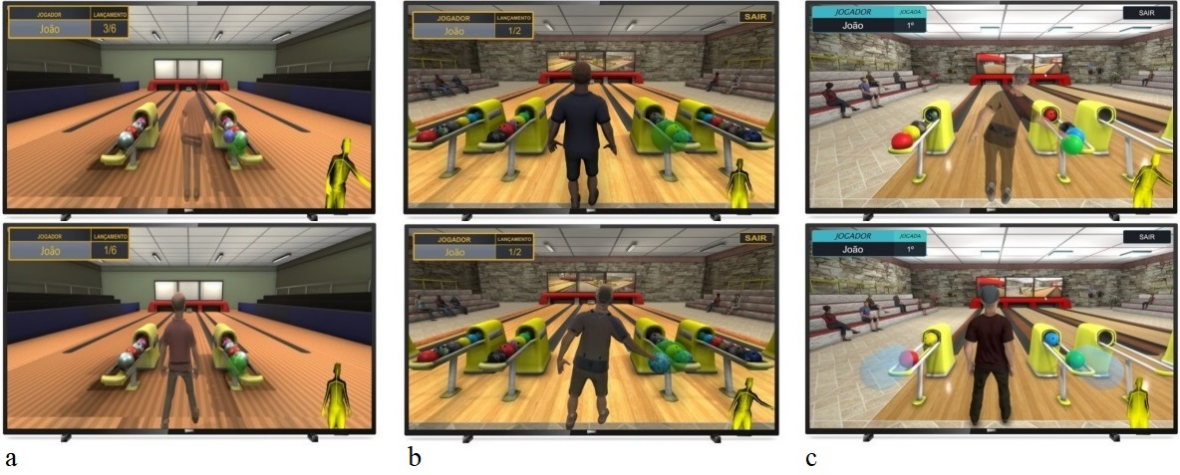
Screenshots showing the evolution of the bowling exergame versions in its (a) first version, (b) intermediate version, and (c) final version.

For the final version, the interface was redesigned with new colors and harmonic proportions, including the option of gesture interaction to go through the menus. Four more character options were added, resulting in 10 different characters. Strike and spares counts were added. At the end of the game, it reported the accuracy and performance of each player based on their score. A complete match history was implemented. New camera animations and unified scenes were added. When there was a strike, confetti effects appeared in the scenario, and the audience cheered. In the menu, we added options for sound volume, sensor distance, and activation of gesture interaction. The lighting system and scripts were optimized, and new background sounds were added. Figure 1 illustrates the evolution of the exergame with the 3 versions used over the testing period.

The final developed exergame, called *Boliche Virtual*, has 2 game modes, one for single players and another to be played in pairs (multiplayer). The Brazilian National Institute of Industrial Property granted us the software copyright registration code BR5120190019121. The game works similar to the real-world 10-pin bowling game, which consists of 10 frames. In each frame, the player has 2 chances to knock down as many pins as possible with the bowling ball. When playing with peers, 2 players are interspersed in each round. Each dropped pin equals 1 point; therefore, the maximum score a player can obtain is 200 points per session. The game’s sequence and scoring system is not entirely similar to the actual bowling system, as it was designed in a more straightforward way to facilitate understanding and gameplay for older players. The total scores for each round, strikes, and spares were stored. At the end of the gaming session, the game announces the winning player and shows the accuracy of each player, ranging from 0% to 100%. Multimedia Appendix 1 provides the screenshots and details of the bowling exergame’s final version.

### Participant Characteristics

At the end of the gaming sessions, 19 participants completed the tests (age: mean 68*.*9, SD 5*.*3 years). In the with peers group, there were 10 participants (7 women and 3 men) with a mean (SD) age of 68*.*8 (5*.*2) years. However, in the alone group, there were 9 participants (6 women and 3 men) with a mean age of 69*.*1 (SD 5*.*8) years. The loss of testing was because of the participants’ loss of interest. All participants were retired, living in the city where the study was performed, and had a higher education level. Although all participants had already used some type of technology, all said that they had never interacted with an exergame before this study. No statistically significant differences were observed between the groups across the baseline demographic variables of gender (*P*=*.*89), age (*P*=*.*90), education level (*P*≥*.*99), or prior knowledge about exergames (*P*≥.99).

### User Testing

Table 1 shows the statistical results of the GPFI score pretest and posttest. Initially, considering the pretest, the sample was homogeneous (*P*=*.*11, *Z*=−1*.*603). Both groups showed significant gains from the exergaming sessions (*P*=*.*005 and *Z*=−2*.*807 for the with peers group; *P*=*.*02 and *Z*=−2*.*273 for the alone group). Comparing the posttest between groups, the with peers group had significantly better results than the alone group (*P*=*.*02, *Z*=−2*.*416).

**Table 1:** General Physical Fitness Index results.

Test	Played with peers (n=10)	Played alone (n=9)	*P* value^a^
Pretest, mean (SD)	40.5 (9.6)	33.9 (7.8)	.11
Posttest, mean (SD)	57.5 (8.7)	44.7 (10.6)	.02
*P* value^b^	.005	.02	N/A^c^

^a^Intergroup comparison using Mann-Whitney *U* test.

^b^Intragroup comparison using Wilcoxon test.

^c^N/A: not applicable.

**Figure 2:**
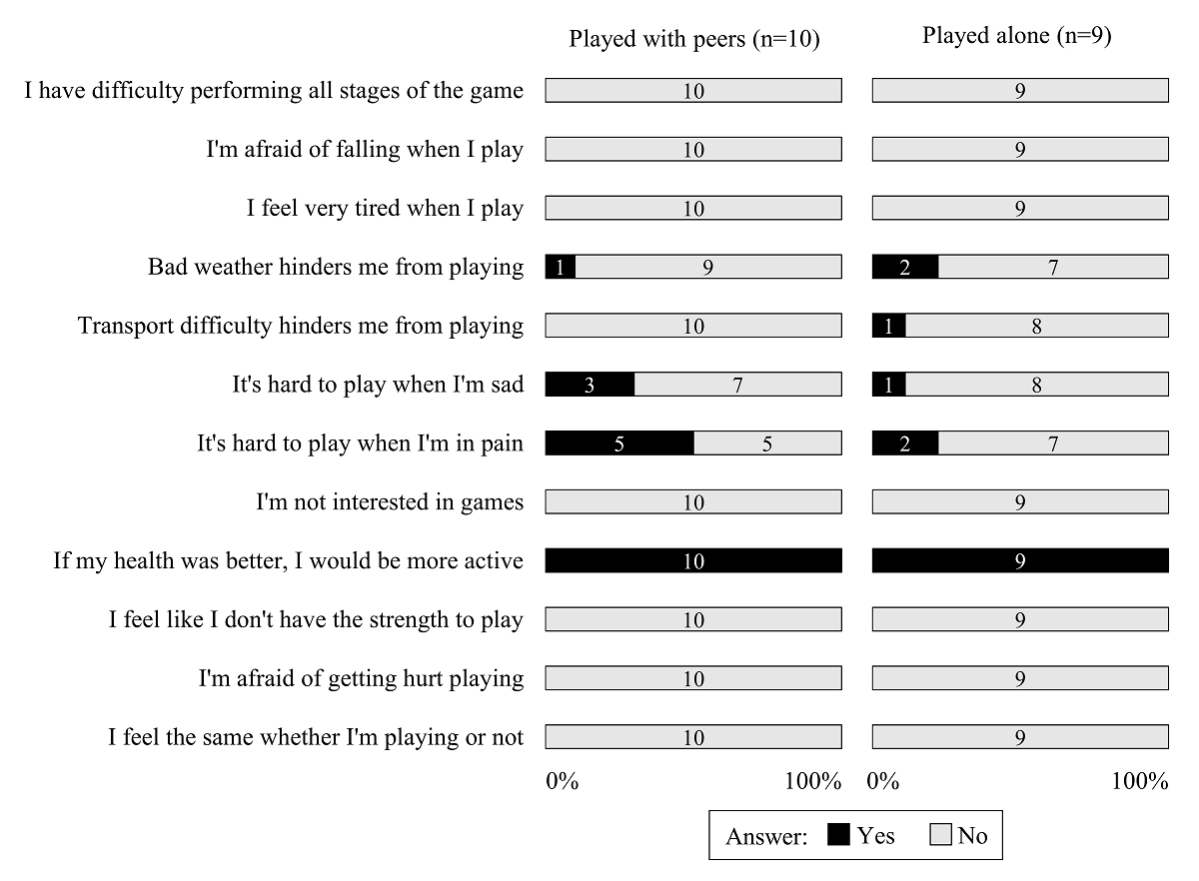
Responses to the adherence statements about barriers when playing the bowling exergame.

**Figure 3:**
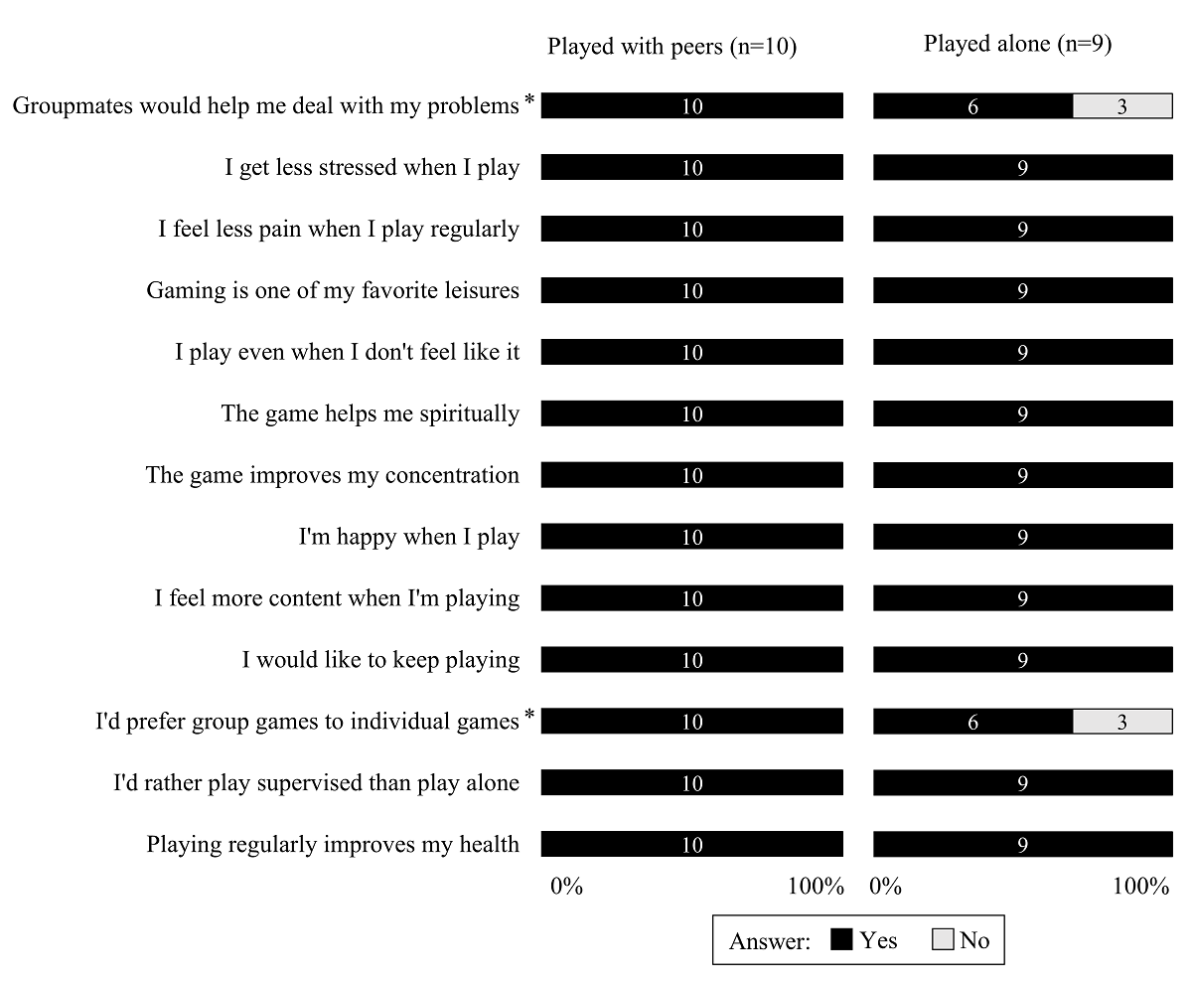
Responses to the adherence statements about motivators when playing the bowling exergame. The asterisk indicates *P*=.04 intergroup.

Figures 2 and 3 show the results of the Physical Exercise Adherence Questionnaire, with statements of barriers and motivators encountered by participants when playing the exergame. As for the motivator statements, the game was motivational for both groups. There was a significant difference between the groups for the statements “Groupmates would help me deal with my problems” (*P*=*.*04) and “I’d prefer group games to individual games” (*P*=*.*04), which had a greater agreement in the with peers group. With regard to the barriers faced by the participants, the with peers group showed higher percentages than the alone group in the questions “It is difficult to play when I am in pain” and “It is difficult to play when I am sad.” In turn, several participants in the alone group reported that “Bad weather hinders me from playing” and “Transport difficulty hinders me from playing.” Finally, all participants in both groups agreed that “If my health was better, I would be more active.” In either group, no participant answered affirmatively to statements about tiredness, difficulty in use, and fear of falling when interacting with the game.

Table 2 shows the results of the GEQ scores and the comparisons with the Mann-Whitney U test, signed intragroup comparisons, with groups as the grouping variable. The only component with a statistically significant difference between groups was sensory and imaginative immersion, which had a higher average score for participants who played with peers. Although nonsignificant, the with peers group obtained a slightly higher average than the alone group in the competence category. In the categories challenge and flow, the values were higher but nonsignificant for the alone group. In the evaluation of participants’ experiences after using the game, the tiredness category had no answers. For both groups, the tension category obtained a lower average of results compared with the other categories. Similarly, there were no answers linked to some aspects of the negative affect category. In contrast, both groups obtained a high average in the positive affect category.

**Table 2:** Mean (SD) scores for the components of the Game Experience Questionnaire.

Module and component^a^		Played with peers (n=10), mean (SD)	Played alone (n=9), mean (SD)	*P* value^b^
**In-game**				
	Competence	3.80 (0.26)	3.72 (0.67)	.55
	Sensory and imaginative immersion	3.90 (0.21)	3.67 (0.25)	.05
	Flow	3.85 (0.24)	3.94 (0.17)	.33
	Tension	0.00 (0.00)	0.11 (0.22)	.13
	Challenge	3.05 (0.90)	3.56 (0.68)	.18
	Negative affect	0.00 (0.00)	0.00 (0.00)	>.99
	Positive affect	4.00 (0.00)	3.89 (0.33)	.29
**Postgame**				
	Positive experience	3.28 (0.08)	3.22 (0.25)	.92
	Negative experience	0.67 (0.00)	0.74 (0.22)	.29
	Tiredness	0.00 (0.00)	0.00 (0.00)	>.99
	Returning to reality	1.07 (0.41)	1.04 (0.26)	.45

^a^Scores can range from 0 to 4.

^b^Mann-Whitney *U* test with signed intragroup comparisons.

## Discussion

### Principal Findings

In this study, either playing alone or with peers, older participants showed significant improvement from pretest to posttest, reaffirming that the practice of exergame is a therapeutic instrument to improve the functional capacity of older people [45]. Nevertheless, the results also suggest that even better outcomes in individuals’ functional capacity and adherence can arise from group-based exergaming, which is similar to the outcomes of group-based conventional exercise [46-48]. Technology is an innovative method to assist changes associated with aging. Supporting older people to participate in physical activity through evaluations offers the added benefit of tracking user activity by monitoring health events and behaviors in real time [49].

Other studies [23,25] suggest that, when comparing traditional exercise with exergaming, both modalities showed more successful results when performed in groups, which can significantly promote social interaction [37]. However, similar experiments [50] have revealed differing outcomes regarding exergaming alone or with peers. Thus, this type of finding deserves further investigation. In this study, we assumed that the participatory co-design process was a key determinant. Notwithstanding, participation in exergames can improve cognitive and physical skills that are directly involved in the functional abilities needed by older people in daily life [51].

Understanding the opportunities, challenges, and training processes that optimize the benefits of exergames has important implications for improving the quality of life and longer-term independence for older people [52]. According to Meekes and Stanmore [27], comprehending the motivation of older people to use exergames can help in the game development process. Thus, throughout the co-design and evaluation period of this study, users were part of the creation process. The contribution that the participant brings to the development of a particular product is crucial to its success, as, by participating in this process, users can see their suggestions being implemented before the product is completed, feeling valued during this process [53,54].

According to Zhang et al [55], the study of familiarity issues among older people is necessary to guide the interaction design of new technologies. Thus, they can be built upon the prior knowledge of older people on real-world interactions, making it easier to apply their existing knowledge and skills to a new domain. Zhang et al [55] also explained that 3 specific design aspects should be followed: representation as similar as possible to the real world to help older people understand the game; manipulation, which indicates how older people manipulate the objects in the exergame; and meaningful design, which includes the stimulation of the older people’s memories and emotions. From a game design perspective, to increase the preference of older people’s attitude toward exergames, it is necessary to consider games with more attractive and comprehensible elements so that the older people can enjoy playing [50,56]. In this sense, a co-design creation process can lead to tailormade challenges that are optimized to the participant’s own preferred level of physical exertion.

In this study, older people were motivated to cooperate directly and indirectly in the game development, similar to what was reported by Chen et al [57] and Kappen et al [58]. We observed that all participants answered *no* to all statements about barriers that were directly related to the game itself. In addition, virtually all participants responded positively to all statements about motivators (241 of 247 individual responses). Furthermore, the participants’ positive experience and interest in their co-designed exergames were evidenced by high scores for complementary components and low scores for derogatory components of the GEQ results.

Other studies highlighted the importance of including the user in the process of development, especially when considering older people [59]. Brox et al [60] reported their results from 3 years of research in the field, providing a user-centered protocol for exergames adapted to the needs of older people. According to the authors, the time devoted to the older people was the main element essential to establish communication and to earn the trust of each one of them. In addition, all types of game elements should be considered during the exergame project, even if they seem too obvious, and particular needs of this target audience must be met during the design process to make exergames more effective [61]. Considering the development of exergames for older people, Chen et al [57] state that playfulness and perceived utility are 2 of the main aspects that affect behavior and the intent to use. According to their study, older people’s acceptance of exergames depends not so much on the fun when playing but on the perceived usefulness of physical and cognitive skills employed on it.

According to Bird et al [62] and Tobaigy et al [63], older people often lack knowledge and perception about how to interact with exergames and how their use can improve health care, until they are exposed to them. In this study, users began to feel comfortable with the game as the sessions progressed, gradually becoming familiar with the way of playing and identifying the benefits of exergaming. The results showed that the interaction with the exergames provided a friendly environment for social interaction, fun, and rehabilitation of the older people in both the groups. When assessing adherence to physical exercise from the motivators, we observed 2 statistically more positive answers in the with peers group to the statements about playing alongside other people. Social aspect was one of the main motivators that led older people to interact with exergames, as mentioned in other studies [10,20,25]. In this context, socializing refers to the interaction between participants, that is, the exchange of experiences facing the same challenge together and competition [64]. Older people can regularly play exergames not only to enhance their health but also to take advantage of the opportunities to socialize with others, which further increases their emotional well-being [50].

Most participants’ experience with the exergames was considered positive. However, the sensory and imaginative immersion achieved a significantly better result for the with peers group, suggesting that the participants in this group became more interested in the game’s story and found it impressive. The fact that playing with a peer is very similar to a real bowling game where participants compete with each other may have contributed to this result, as the exergame may have an effect similar to the conventional bowling game [65]. Usually, exergames use an avatar to represent a player in the virtual world. Studies suggest that the avatar’s appearance may be a key factor that influences player behavior [66]. When choosing an avatar reflecting themselves, players have higher perceived game interactivity. This can be a powerful motivator, leading to higher engagement during gameplay. In this study, female participants reported that they felt more attached to the game when a female character was available to play. In addition, participants felt more captivated by the game during the final sessions when improved characters and new elements were added.

### Limitations

Some limitations of this study include the fact that we only explored competition instead of collaboration among the user participants and the fact that the study had a small sample size because of dropouts, which makes it difficult to generalize the results. With regard to the study design, nonblinding may have negatively influenced the findings, although the groups were homogeneous in age and functional capacity. We had to rely on the participants’ claims about not performing other physical exercises throughout the study, thus avoiding confounders. In addition, the bowling game has specific movements; other types of games may use different ones and therefore may not elicit the same outcomes. During the sessions, participants kept interacting with the game even when it was not completely ready in its final version. If participants had only interacted with the final version of the game, the results might have been different. In addition, participants attended the same club and became even more familiar with their peers throughout the sessions. In this sense, playing with stranger peers or in a smaller number of sessions would not necessarily lead to the same findings. Only older people who already practiced some physical activity participated in the experiment; therefore, the results do not necessarily apply in an equivalent way to older people with sedentary habits.

### Conclusions

Exergames as motivators of physical activity have been increasingly seen as promising and encouraging tools. The findings of this study suggest that exergaming, either alone or with peers, leads to a statistically significant improvement in functional capacity. However, evidence suggests that an exergame is more attractive and successful when participants play it with peers. With regard to adherence to physical exercise, both groups showed interest in playing alongside other people. Another contribution of this study was the creation of the exergame *Boliche Virtual*. Our approach offered a collective experience with the use of technology, placing the user at the center of the game design process. This approach may have influenced the motivation and adherence of participants and, consequently, elicited better health outcomes. Therefore, co-design and testing using a participatory and iterative process might foster positive results.

More research applying this design approach is needed to understand the opportunities, challenges, and training processes that can optimize exergames to improve the quality of life and long-term independence of older people. In future works, we intend to perform new tests with a larger number of participants. Other aspects related to player experience can be assessed, such as decision making. We also suggest the long-term monitoring of the participants to verify the maintenance of results and to further investigate the differences in exergaming with peers.

## Appendix

Multimedia Appendix 1 Screenshots and details of the bowling exergame's final version.

## Abbreviations

3D: 3-dimensional
GEQ: Game Experience Questionnaire
GPFI: General Physical Fitness Index
