# Supplementary material for: A Bowling Exergame to Improve Functional Capacity in Older Adults: Co-Design, Development, and Testing to Compare the Progress of Playing Alone Versus Playing With Peers
Source: JMIR Serious Games. 2021 Jan 29;9(1):e23423. doi: 10.2196/23423 (PMC7880815; doi:10.2196/23423)
Supplement: Multimedia Appendix 1 [file games_v9i1e23423_app1.pdf]

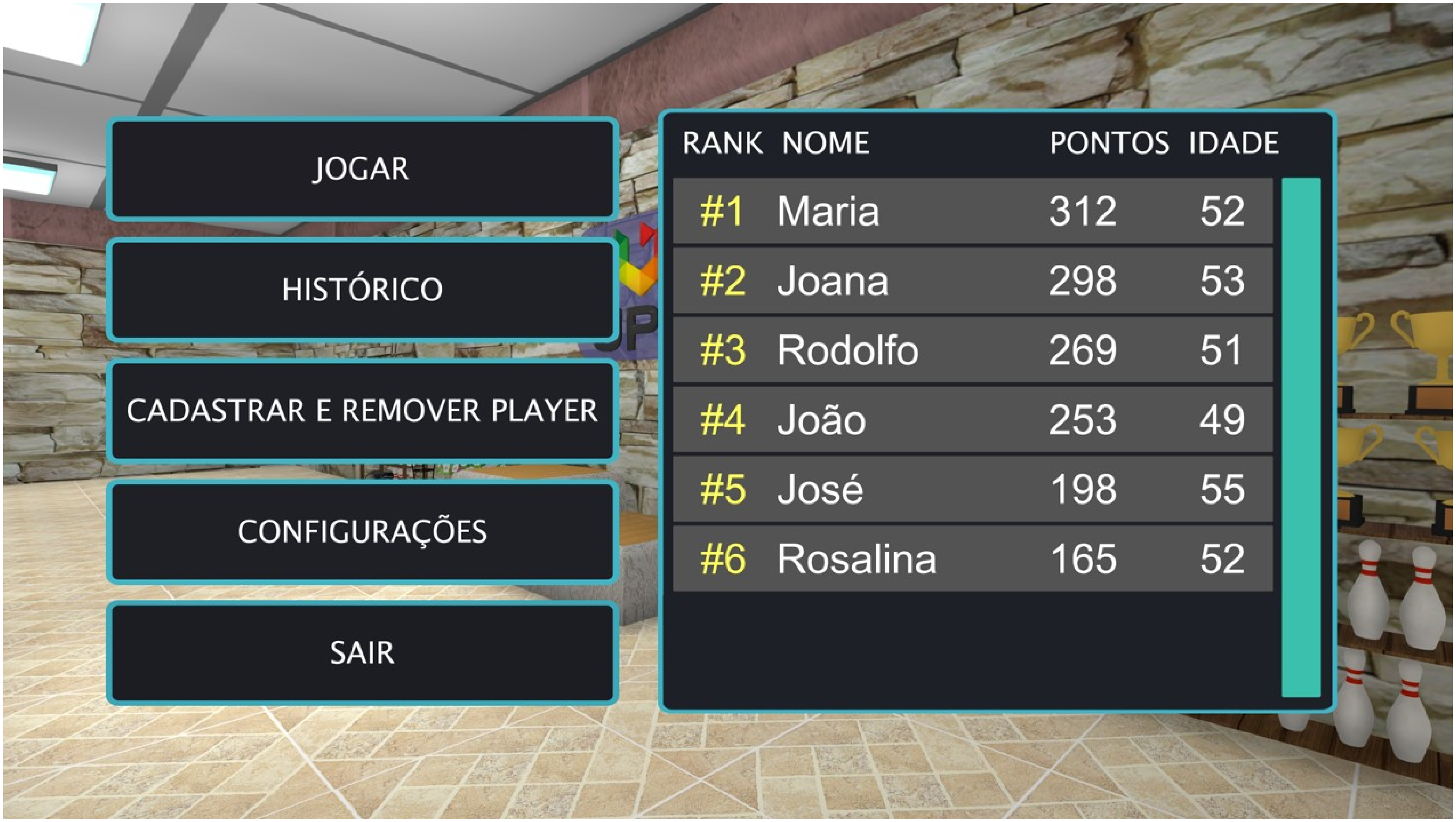

Opening screen. It shows the ranking of the scores of all registered players. On the right side, there is also a ranking position, name, total points, and age (optional) of each player. The left side shows the menu with five buttons that hold the following options: Play, history, register and remove player, settings, and exit.

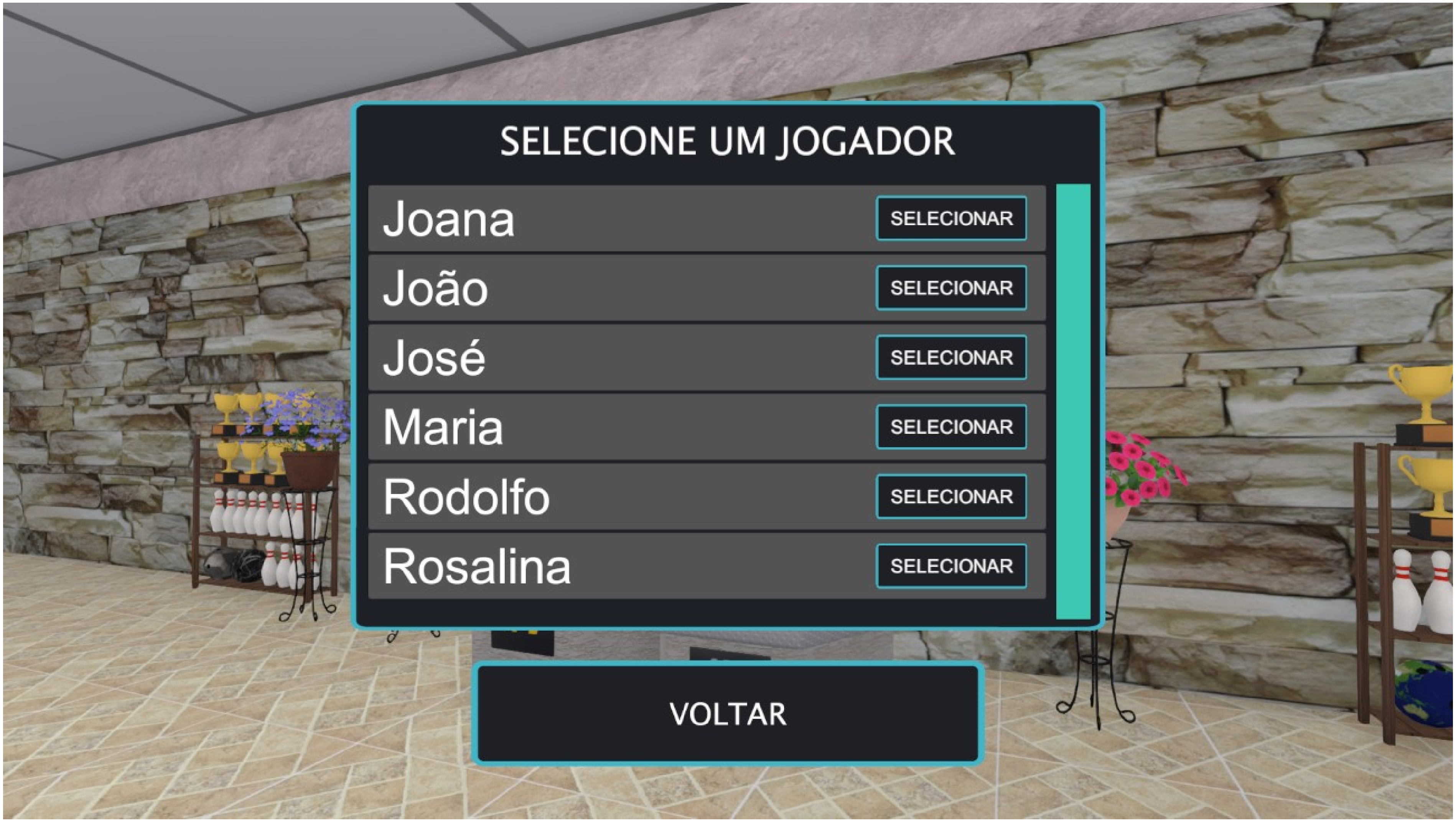

Player options before entering a game match in the multi-player mode. There are two buttons next to each player's name to select who will be player 1 and who will be player 2. There is another button to return to the mode selection screen at the bottom of the player selection.

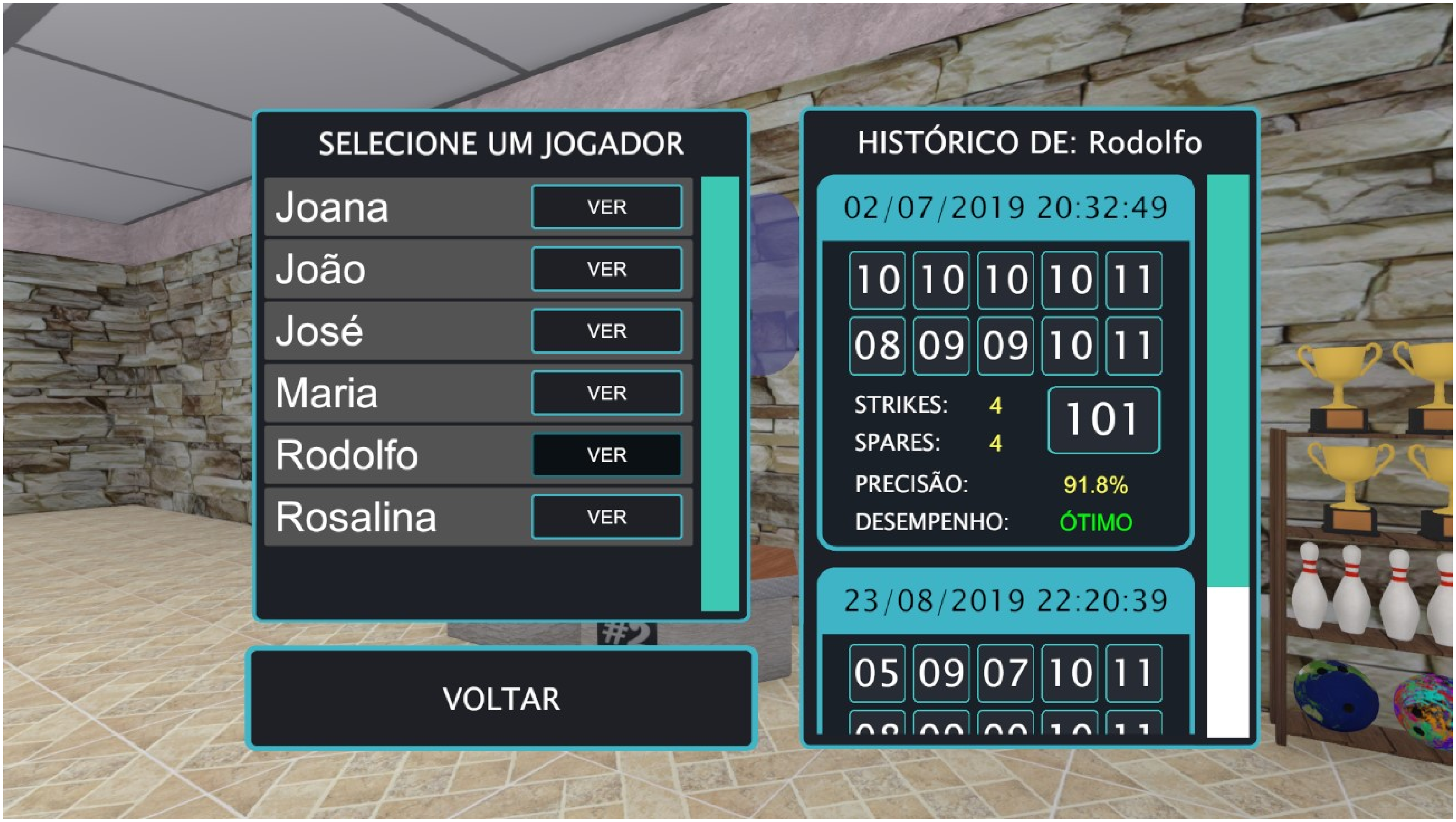

Player selection to view match history. The menu has an option to view each player's history. The screen's left side shows a list of the available players. Each name has a button on the right side so that the name can be selected. The right side shows the player's name with a score per match and the results of the matches played, sorted by the match date. There is another button to return to the mode selection screen at the bottom of the player selection on the left side.

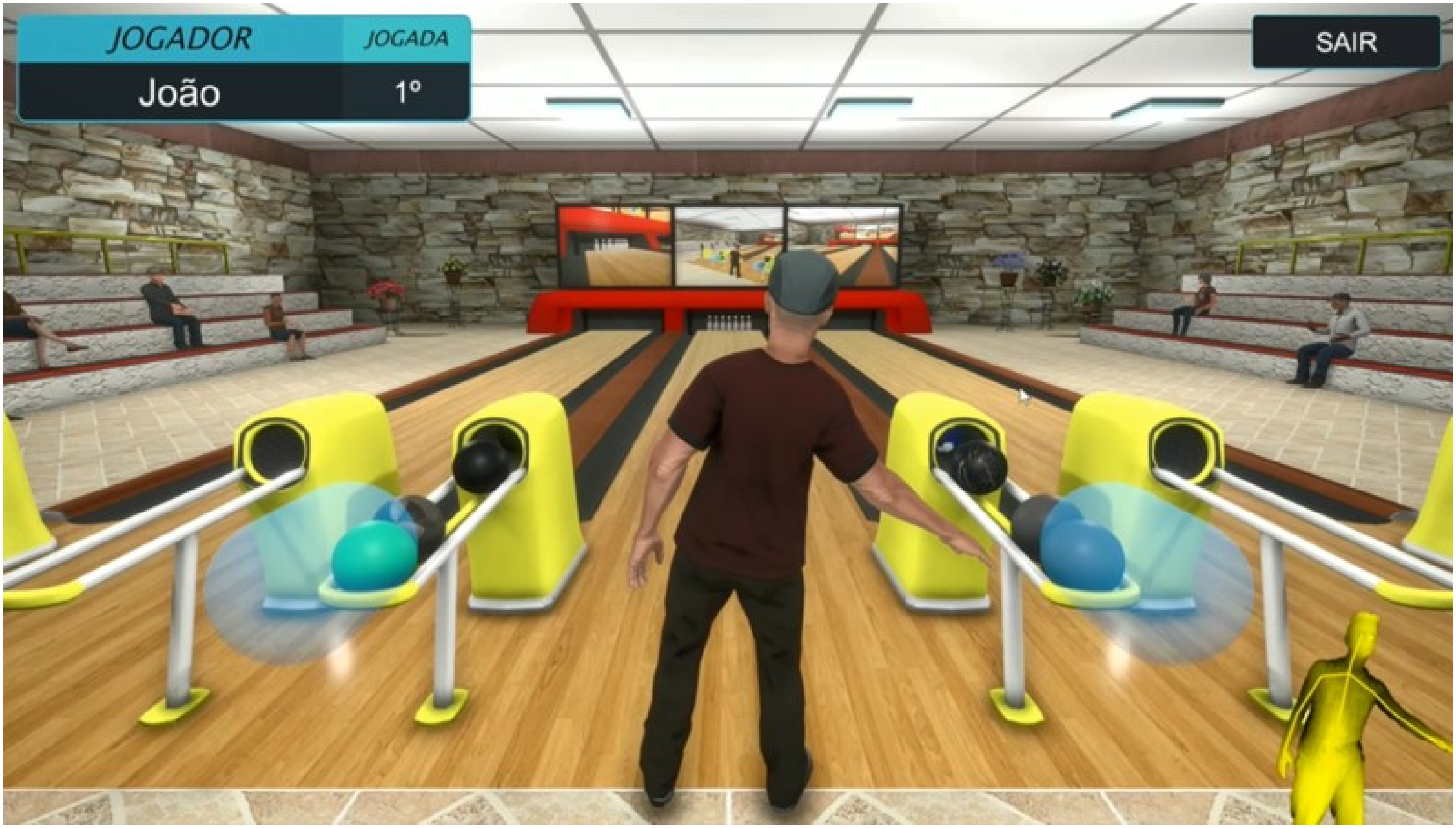

Main game activity. The left panel exhibits the name of the current player and which is the current throw. In this screen, the player is reaching for the ball to perform his play. The bottom right shows the player's movements as captured by the Kinect motion sensor. The button on the top right can end the match and return to the main screen.

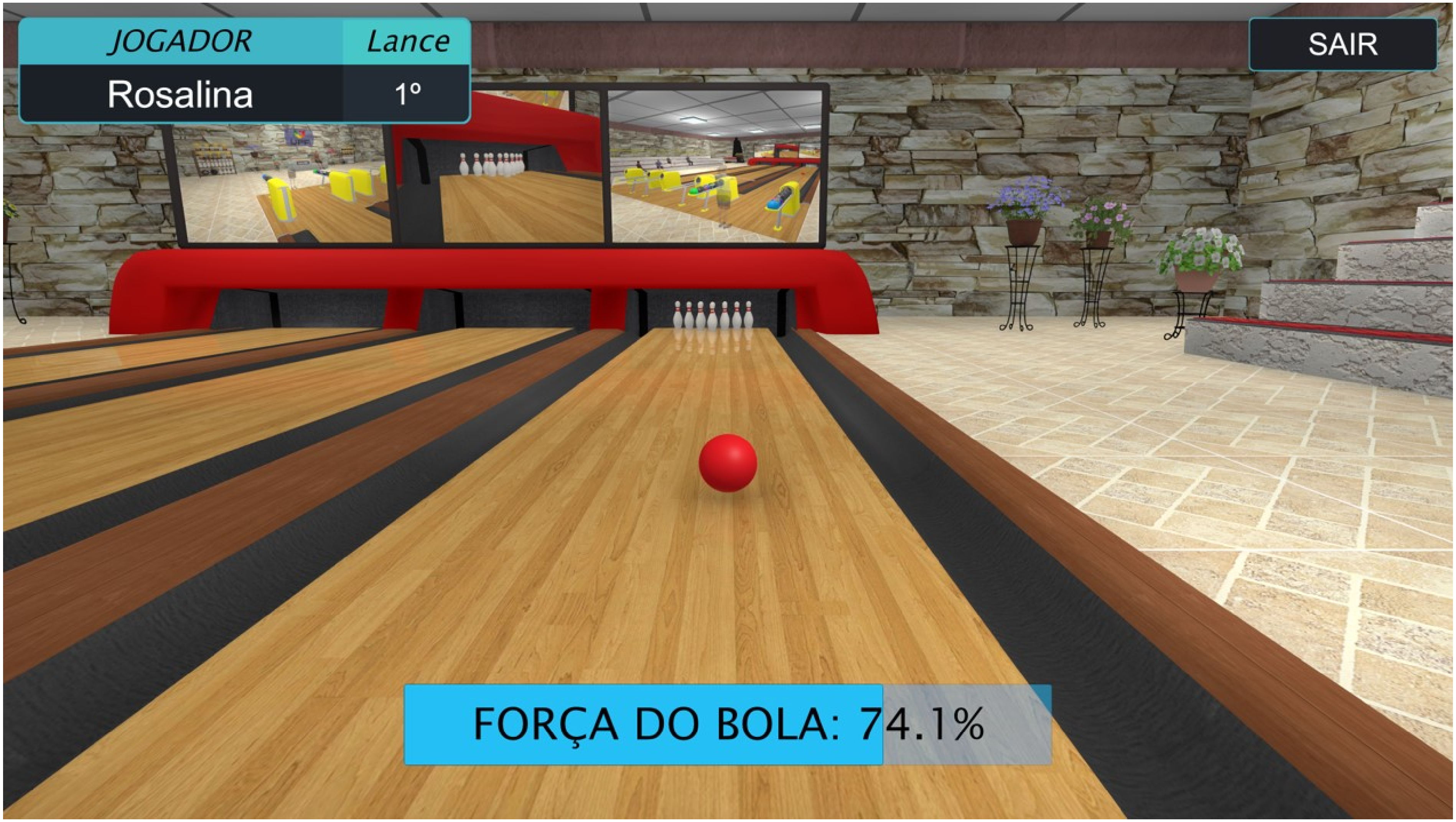

Ball rolling towards the pins during a throw and a blue progress bar showing the ball strength that the player applied during the movement.

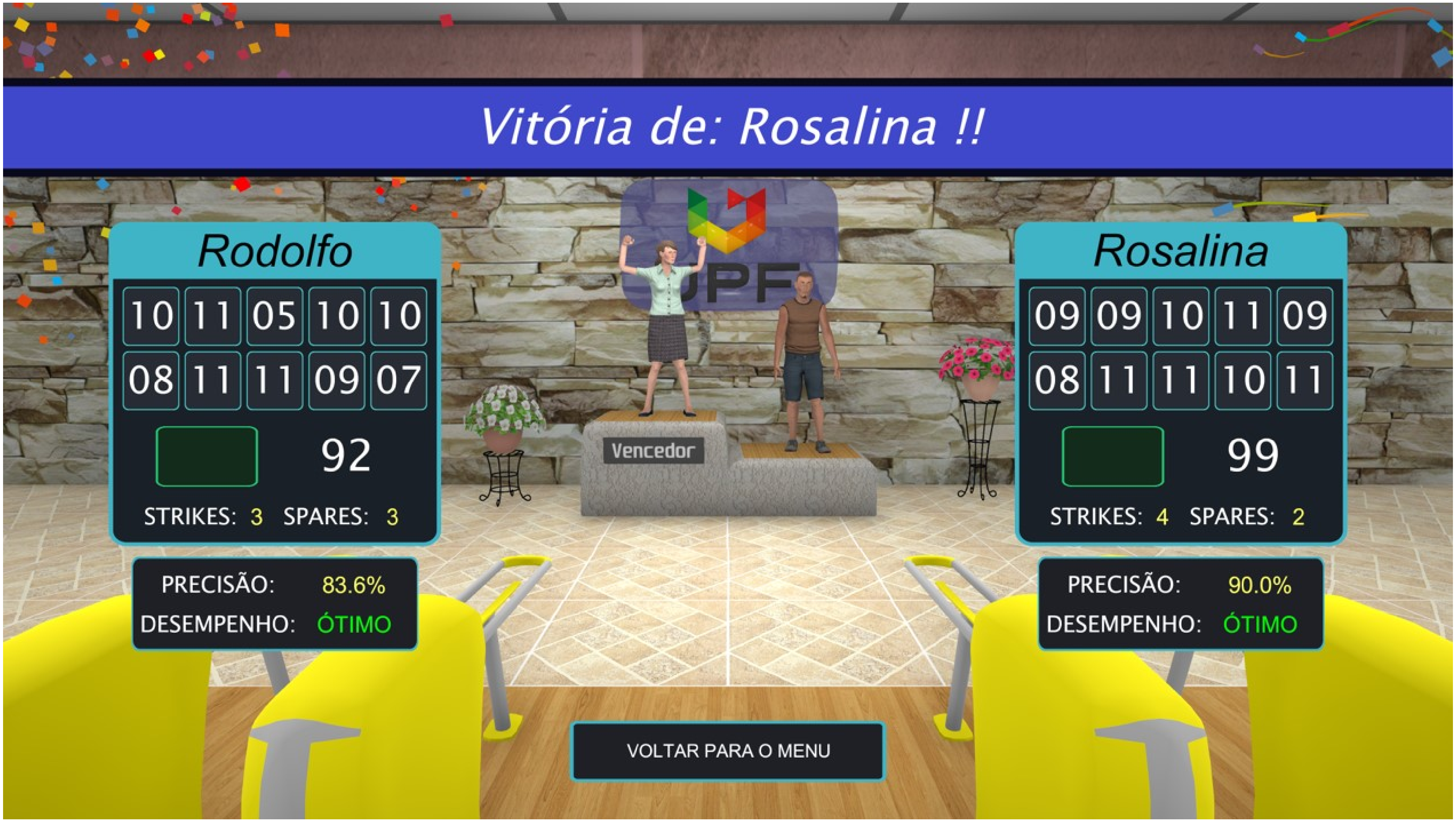

Match-winner standing on a podium celebrating victory. Two information boxes show each player round's score, total score, the number of strikes and spares, accuracy (0% to 100%), and player's overall performance (weak, medium, good, or excellent).
